# Skewfoot Deformity: State of the Art

**DOI:** 10.3390/children12060760

**Published:** 2025-06-12

**Authors:** Antonio Mazzotti, Federico Sgubbi, Alberto Arceri, Gianmarco Di Paola, Elena Artioli, Simone Ottavio Zielli, Lorenzo Marcucci, Nicola Guindani, Cesare Faldini, Maurizio De Pellegrin

**Affiliations:** 1 1st Orthopaedics and Traumatologic Clinic, IRCCS Istituto Ortopedico Rizzoli, 40136 Bologna, Italy; antonio.mazzotti@ior.it (A.M.); federico.sgubbi@ior.it (F.S.); gianmarco.dipaola@ior.it (G.D.P.); simoneottavio.zielli@ior.it (S.O.Z.); cesare.faldini@ior.it (C.F.); 2 Department of Biomedical and Neuromotor Sciences (DIBINEM), Alma Mater Studiorum University of Bologna, 40123 Bologna, Italy; 3 Orthopedic Residency Program, University of Verona, 37134 Verona, Italy; lorenzo.marcucci@studenti.univr.it; 4 Orthopaedics and Traumatology, Papa Giovanni XXIII, 24127 Bergamo, Italy; nguindani@asst-pg23.it (N.G.); depellegrin1956@gmail.com (M.D.P.); 5 Pediatric Orthopedic Unit, Piccole Figlie Hospital, 43125 Parma, Italy

**Keywords:** skewfoot, skew foot, Z-foot, S-shaped foot, serpentine foot

## Abstract

**Background**: Skewfoot, also known as Z-foot, is a rare and complex deformity characterized by a combination of forefoot adduction and hindfoot valgus, resulting in a “Z” shape. Due to its rarity, diagnostic criteria and standardized treatment guidelines are lacking. This scoping review aims to systematically map and summarize the current knowledge regarding skewfoot. **Methods**: A comprehensive literature search of the PubMed, Cochrane Library, and Scopus databases was conducted to identify relevant articles. Patient-specific data were meticulously extracted from eligible studies and analyzed in detail. **Results**: A total of 12 studies met the inclusion criteria. Each study was independently reviewed, and data on epidemiology, etiology, clinical presentation, imaging assessment, and treatment options were extracted. **Conclusions**: The true incidence of skewfoot remains unknown. Etiology is likely multifactorial, often associated with systemic and neurological disorders. Skewfoot management ranges from conservative approaches to surgery. A medial cuneiform opening wedge osteotomy is the most used technique; however, the frequent need for additional procedures emphasizes the complexity of the deformity and the importance of a personalized approach.

## 1. Introduction

Skewfoot is a complex deformity characterized by a combination of forefoot adduction and hindfoot valgus [1].

The terminology varies, and the condition is also referred to as Serpentine foot [2] S-shaped foot [3], Z-foot deformity [4,5], or *Serpentinenfuß* [6]. Initially described by Henke in 1863 [6], it was Peabody and Muro who highlighted the necessity to treat it differently from other similar deformities, such as metatarsus adductus [2].

The exact incidence of skewfoot remains poorly established and is considered rare, with no evidence of gender predominance. Its prevalence may also be underestimated due to diagnostic challenges [7], particularly in distinguishing it from simple metatarsus adductus, which exclusively affects the forefoot [1,5,8,9].

Skewfoot recognizes different etiology, either congenital or acquired [1]. The diagnosis is primarily clinical [8]. Imaging is crucial for a comprehensive evaluation, typically involving weight-bearing radiographs [1], complemented by second-level imaging techniques such as Computed Tomography (CT) scans, Magnetic Resonance Imaging (MRI), or ultrasound [9,10,11,12,13]. Due to variable clinical presentation, various classification systems have been proposed [14,15,16].

Both conservative and surgical approaches have been described; however, the literature lacks a standardized treatment algorithm [1,8,17].

This scoping review aims to consolidate the current knowledge on skewfoot deformity, addressing its epidemiology, etiology, clinical presentation, imaging evaluation, and treatment options.

## 2. Materials and Methods

Given the complexity and inconsistency of this topic, with heterogeneous and widely dispersed evidence, a scoping review approach was selected to comprehensively map the existing literature, in order to identify key concepts, theoretical frameworks, sources of evidence, and research gaps. This method facilitates a broad examination of the literature without the restrictions of a systematic review.

The recommended methodology for a scoping review was first developed by Arksey and O’Malley [18], with subsequent refinements by Levac et al. and Peters et al. from the Joanna Briggs Institute [19,20]. For this scoping review, the guidelines and checklist proposed by Tricco et al. [21] were followed.

### 2.1. Identification of Relevant Studies

A comprehensive computer-based search was conducted on 24 December 2024, across three databases, PubMed, Scopus, and Cochrane Library, to identify all published articles related to Skewfoot, including data for each patient involved in the study. For this purpose, combinations of the following keywords were utilized: “skewfoot”, “skew foot”, “z-foot”, “s-shaped foot”, “z-shaped foot”,” hindfoot valgus”, and “forefoot adduction”. The Boolean operators ‘and’ and ‘or’ were used to combine these terms where relevant to produce final search strings.

Additionally, grey literature sources were explored, including Google Scholar and direct communication with experts in the field. Grey literature refers to research that is either unpublished or disseminated through non-commercial sources, such as dissertations, conference proceedings, and government reports. Reports were assessed for eligibility if the studies were directly related to skewfoot.

Exclusion criteria encompassed articles in which skewfoot was not the primary focus; non-English-written publications; biomechanical or cadaveric studies; and brief communications, letters to the editor, quiz cases, reviews, and meta-analyses. No restrictions were applied based on publication year. Case reports were included.

### 2.2. Study Selection

Following the removal of duplicate entries, two authors (F.S. and G.D.P.) independently reviewed the titles and abstracts of the remaining papers to determine their eligibility based on selection criteria. Full-text versions of the relevant articles were then obtained for further assessment. Any disagreements regarding article inclusion were resolved by consulting the senior author (A.M.). To systematically review the relevant literature for this comprehensive analysis, a structured search was designed using the Population, Concept, and Context (PCC) framework, according to Preferred Reporting Items for Systematic Reviews and Meta-Analyses extension for Scoping Reviews (PRISMA-ScR) guidelines [21].

The “Population” of interest included any patients affected by skewfoot. The “Concept” focused on epidemiology, etiology, diagnosis, clinical, and radiological investigation and various conservative and surgical management strategies, particularly emphasizing procedural differences between surgical and nonsurgical approaches. No limitations about the “Context” group were required and no date restrictions were imposed. Studies were included if they contained at least one case of skewfoot, even when the primary focus of the article was on other foot deformities, as long as patient-level data on skewfoot could be clearly extracted.

Study characteristics retrieved from the studies encompassed the author, publication year, study design, and Level Of Evidence (LOE).

### 2.3. Data Extraction

Two reviewers (A.A and G.D.P.) independently extracted data from the included studies. Data collection and organization were facilitated using Microsoft Excel 360 (Microsoft Corporation, Redmond, WA, USA) for Windows 11.

The following data were extracted from selected studies:

- Epidemiology;
- Etiology;
- Clinical presentation;
- Imaging assessment;
- Treatment options.

### 2.4. Collating, Summarizing and Reporting Results

A qualitative thematic approach to the reporting and summarizing of results was employed throughout. This method uses identification of key themes found in the literature and summarizing results, according to these different themes, using tables and narrative review. Such an approach is commonly used when performing scoping reviews as it provides reviewers the ability to map and summarize the literature according to these important themes, as is congruent with the purpose of a scoping review.

## 3. Results

### 3.1. Search Results

**Figure 1:**
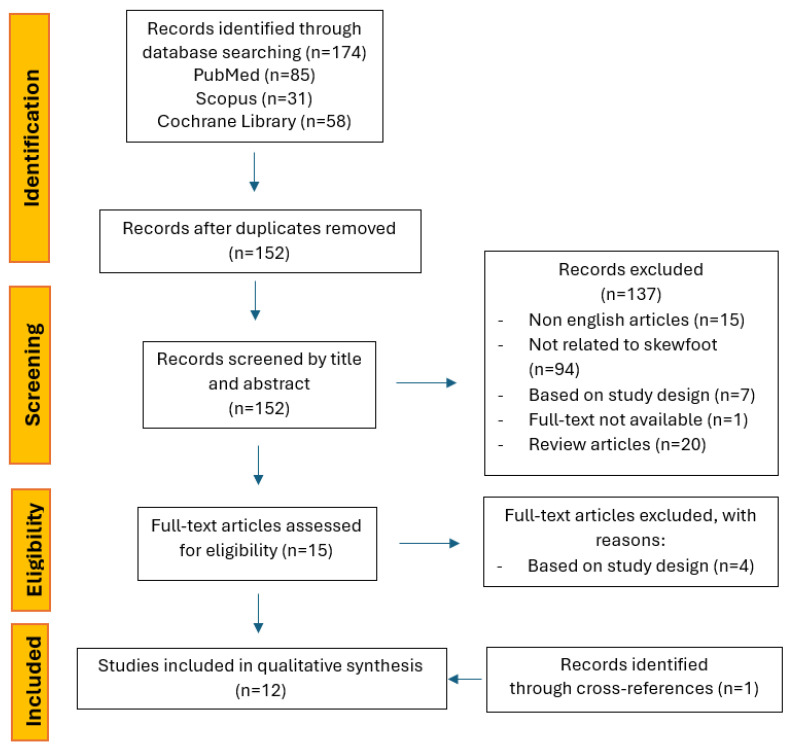
Flowchart of review process by PRISMA.

The initial literature search retrieved a total of 174 articles, including 85 studies from PubMed, 58 from the Cochrane Library, and 31 from Scopus. After removing duplicates, the titles and abstracts of the 152 remaining studies were screened to select those eligible for inclusion in the review. Of these, 141 studies were excluded with the reasons shown in the PRISMA flow diagram (Figure 1). The remaining 15 full-text articles were reviewed for further evaluation of eligibility. One additional study was identified through cross-referencing during the full-text screening process. No relevant reports were found from the grey literature. A total of 12 studies met the inclusion criteria and were included in the scoping review (Table 1).

### 3.2. Level of Evidence

Of the included studies, six [13,15,22,23,24,29] reported only one patient with skewfoot and were thus considered case reports (LOE V), according to the Oxford Centre for Evidence-Based Medicine 2011 Levels of Evidence. The remaining six studies [16,25,26,27,28,30] were considered retrospective case series and classified as LOE IV. This reclassification was performed after a detailed review of each study’s reported patient numbers and aligns with current guidelines. The articles spanned from 1986 to 2022 (Table 1).

### 3.3. Extracted Data

#### 3.3.1. Epidemiology

A total of 78 cases of skewfoot were included in the review. Eleven papers [13,15,16,23,24,25,26,27,28,29,30] reported the ages of patients, with a mean age of 6.31 years (range: 1 year 6 months–19 years). Only seven studies [13,15,23,24,26,29,30] reported the sex of the participants, comprising five females (42%) and seven males (58%) (Table 1).

#### 3.3.2. Etiology

Different etiologies are reported in Table 2.

In 55 feet (71%), the etiology was not explicitly reported [16,22,23,24,25,27,30].

Etiology information was provided in 23 cases (29%) [13,15,26,28,29].

The most frequently reported etiology was congenital, either associated with systemic or neurogenic disorders. Among them, skewfoot associated with systemic disorders included three cases of osteogenesis imperfecta (4%) [26], 3 cases of Angelman’s syndrome (4%) [29], and 2 cases of Freeman–Sheldon syndrome (3%) [15]. Regarding congenital cases associated with neurogenic conditions, four cases (5%) were documented with myelomeningocele [28].

Idiopathic congenital skewfoot was explicitly reported in only two cases (3%) [28].

An iatrogenic etiology was identified in one case (1%), as a result of surgical treatment for clubfoot, which included Achilles tendon release, posteromedial release, and triple fusion [13].

#### 3.3.3. Clinical Presentation

Six studies reported a clinical examination on their patients [13,15,16,28,29,30] covering a total of 51 feet (65%).

The most common clinical characteristics were hindfoot valgus with forefoot adduction, reported in all 51 cases (65%) [13,15,16,28,29,30]. Among these, seventeen cases (22%) also presented with lateral subluxation of the talonavicular joint, as reported in two papers [15,16], six cases (8%) also exhibited retraction of the Achilles tendon [28], four cases (5%) also reported midfoot supination [13,29], and one case (1%) presented with medially skewed toes with fixed flexion as a sequela of clubfoot [13].

Clinical presentations are reported in Table 3.

#### 3.3.4. Imaging Presentation

Imaging evaluation was performed using X-rays in all cases [13,15,16,22,23,24,25,26,27,28,29,30], with only one study associated with CT scan assessment [15].

Imaging presentations are summarized in Table 3.

Fifteen cases (19%) did not include detailed radiological findings, despite imaging being performed [22,23,24,25,30].

Skewfoot was analyzed and documented using X-rays in two standard projections, Dorso-Plantar (DP) and Latero-Lateral (LL), in 55 cases (71%).

Specifically, in the DP projections, the following anatomopathological findings were reported:

• Increased talo-first metatarsal angle, reported in 48 feet (62%), with a mean value of 17.8° (range: 4°–36°) [16,27].
• Increased talocalcaneal angle (Kite’s angle) in 32 cases (41%) [13,26,27,29,30], with a mean angle of 39° (range 21°–80°) [27,29].
• Medial angulation of the base of the three medial metatarsals in 17 cases (22%) [27].
• Lateral subluxation of the navicular on the talus in 14 cases (18%) [26,29,30].
• Forefoot adduction in seven cases (9%) [13,26,29].
• Midfoot supination in one case (1%) [13].

Findings in the LL projections included the following:

• Increased talocalcaneal angle (Kite’s angle) in 30 cases (38%) [15,26,27,30], with a mean value of 54° (range 28°–63°) [27].
• Increased talo-first metatarsal angle in six cases (8%), with a mean value of 27° (range: 3°–52°) [28].
• Increased talo-horizontal angle in six cases (8%), with a mean value of 36° (range: 25°–65°) [28].
• Reduced calcaneal pitch in six cases (8%), with a mean value of 5° (range: −14° to 11°) [28].
• Dorsiflexion of the talonavicular joint in five feet (6%) [15,26].
• Plantar flexion of the tarsometatarsal joints in two feet (3%) [15].
• Flattening of the longitudinal arch in one foot (1%) [13].
• CT scans were performed in only two feet (2%); to characterize the deformity, a line was drawn to the head of the talus and through the body of the talus, forming a Z shape [15].

#### 3.3.5. Treatment Options

Treatment options are reported in Table 4.

Both conservative and surgical approaches were described.

In the case of asymptomatic patients (seven cases—9%), normal footwear was advised. [16,26].

In pediatric patients (mean age of 2.5 years; range of 0.5–2.75) gentle manipulations, stretching, and serial casting in corrective positions were performed in 33 skewfoot cases (42%) [15,16,29,30], followed by the use of Denis Browne splints in 27 cases (35%) [16,30], outflare shoes in eight cases (10%) [30], and straight shoes in three cases (4%) [29]. The serial casting technique consists of holding the hindfoot in varus with lateral pressure on the talus and metatarsals, using the cuboid as a fulcrum [30,31].

Surgical treatment involved both bone and soft tissue procedures [13,16,22,23,24,25,27,28,29,30]. Regarding soft tissue, the most common procedure was medial capsulotomy of the navicular–cuneiform and cuneiform–first metatarsal joints, combined with abductor hallucis lengthening performed in 17 cases (22%) with a mean patient age of 3.6 years (range: 0.75–5.5) [27]. Extensive posteromedial release, including talocalcaneal, talonavicular, and navicular–cuneiform capsulotomies, along with Achilles and tibialis posterior tendon lengthening, was performed in six feet (8%) [30].

Bone procedures, often combined with soft tissue ones, most commonly involved medial cuneiform opening wedging osteotomies, performed in 13 cases (17%) with a mean age of 7.5 years (range 3.8–15), refs. [22,23,24,25,28]. These were variably associated with cuboid closing wedge osteotomy in five cases (6%) (mean age: 7.4 years, range: 5.5–15) [23,25]; with calcaneal lengthening and Achilles tendon lengthening in six cases (8%) (mean age: 8.3 years, range: 5.75–13) [28]; with talonavicular plication and tibialis posterior advancement in four cases (5%) [28]; or with calcaneal osteotomy, peroneus transfer, plantar fascia release, and tibialis posterior advancement in one case (1%) [22]. Additionally, tibialis anterior re-tensioning was reported in one case (1%) in a 3.8-year-old patient [24].

Furthermore, calcaneal-cuboid closing wedge fusion, multiple metatarsal osteotomies, and plantar fascia release were described in two feet (3%) in a 5-year-old patient [29].

One study reported a calcaneal dome lateral opening wedge osteotomy, dorsiflexing osteotomy of the first metatarsal with first metatarsophalangeal joint fusion, flexor tenotomies of the lesser toes, and transfixation with K-wires for one (1%) case [13].

Fusion of the subtalar joint using the Grice technique was reported in six cases (8%) [30], as well as triple fusion in one case (1%) [29], both following the failure of previous treatments.

## 4. Discussion

This scoping review aims to consolidate current knowledge regarding skewfoot.

Skewfoot seems a rare condition [2,32]; the real incidence is unknown and underestimated due to the challenging diagnosis [5,9,32]. This review reported a male-to-female ratio of 7:5, while the age at diagnosis varied widely, from the first months in congenital cases to adolescence in iatrogenic and idiopathic cases.

The etiology of skewfoot is likely multifactorial. Napiontek [8] developed a clinical classification that is useful for distinguishing the various etiologies. According to this classification, four types of skewfoot can be identified, encompassing the main etiology reported in the literature: congenital idiopathic, congenital associated with syndromes or systemic disorders, neurogenic, and iatrogenic.

The pathogenesis of congenital idiopathic skewfoot remains debated. A widely cited theory is that in untreated metatarsus varus, the medial contact of the foot with the ground during gait induces a lateral deviation of the hindfoot [14,30,32]. The deformity force leads to soft tissue imbalance, with the apex of the deformity centred on the cuneiforms [14]. However, this does not account for cases diagnosed prior to ambulation, where intrauterine positioning has been proposed as a contributing factor [1,33]. Anatomical anomalies in the tibialis anterior—such as distal [2,33] or atypical insertions, aberrant tendon pathways, and shortened medial cuneiform [8,14]—have also been observed, though inconsistently confirmed intraoperatively [34,35].

Skewfoot is occasionally associated with syndromes or systemic disorders (e.g., Freeman–Sheldon syndrome, osteogenesis imperfecta, Larsen syndrome), suggesting a possible genetic component [8,15,26,36,37], although hereditary transmission has not been demonstrated. Neurological disorders, including cerebral palsy and myelomeningocele, may present with secondary skewfoot [1,28]. Iatrogenic cases may be due to improper casting techniques [32,38,39] and, less commonly, due to surgical over-release during the correction of congenital talipes equinovarus [8,40] in cases of complete subtalar capsulotomy or sections of talocalcaneal ligament [40].

Regarding clinical presentation, skewfoot was originally described as an adduction deviation of the metatarsus on the tarsus, associated with internal rotation of the talus and valgus deviation of the calcaneus [2]. The current literature agrees with this presentation [1,7,41] but emphasizes the difficulty of differentiating this condition from simple metatarsus adductus, particularly in the presence of abundant adipose tissue in the infant foot. Diagnosis may be missed due to the opposite directions of the forefoot and hindfoot deformities, which may mask the deformity [1]. Torsional alterations in the lower extremities may further contribute to a deceptively normal appearance [41]. Midfoot alignment in skewfoot may range from normal alignment [32,41] to midfoot abduction and lateral translation [14]. Midfoot abduction may be part of the collapse of the plantar arch in relation to hindfoot valgus. This distinction allows classification into simple skewfoot (normal midfoot) and complex skewfoot (lateral deviation–abduction of the midfoot) [38].

Achilles tendon retraction is usually a secondary adaptation to hindfoot valgus [30]. Clinical presentation also varies by age and comorbidities: in children under three, the deformity is typically flexible, becoming more rigid with weight bearing [30], while syndromic cases often exhibit marked rigidity [8].

For diagnostic purposes, standard weight-bearing radiographs providing essential information on skeletal alignment, joint subluxation, and the severity of the deformity. Weight-bearing DP radiographs typically reveal forefoot adduction, midfoot abduction, and hindfoot valgus, contributing to the characteristic serpentine appearance of the deformity [1]. This parameter is particularly useful for assessing the forefoot–hindfoot relationship in cases where the navicular has not yet ossified [16]. Although an increased talo-first metatarsal angle was frequently reported, the presence of two angular deformities in opposite directions can create a potentially normal angle that does not necessarily exclude the diagnosis [28].

Medial angulation of the bases of the three medial metatarsals has been proposed as a marker of forefoot orientation, as described by Asirvatham et al. [27] Additionally, the metatarsus adductus angle, formed by a line parallel to the second metatarsal and a line perpendicular to the bisector of the midfoot or the second cuneiform, can be used to assess forefoot alignment. An angle > 21° using the first method or 24° using the second is considered pathological [42]. However, this measurement is infrequently reported in the literature. Another radiographic indicator of this pathology is the longitudinal alignment of the first metatarsal, the three cuneiform bones, and the cuboid [43,44]. Moreover, an increased DP talo-calcaneal angle (Kite’s angle) can be observed [16,27].

DP radiographs also allow us to differentiate simple and complex deformities based on whether the longitudinal axis of the calcaneus bisects the cuboid and the base of the fourth metatarsal (simple skewfoot) or if the cuboid is lateral to this axis (complex skewfoot) [9,38].

On an LL radiograph, an increased talo-calcaneal angle (Kite’s angle), talo-first metatarsal angle, talo-horizontal angle, and reduced calcaneal pitch were observed. These parameters were associated with hindfoot valgus [45,46]. Currently, no universally accepted radiographic parameters exist to precisely quantify the degree of deformity.

MRI and CT scans are useful for quantifying subluxation of the navicular bone [11] [12] and for measuring the lateral displacement of the base of the first metatarsal relative to the main talar axis [11]. Most of the essential information required can be obtained from standard radiographs, even in cases of incomplete ossification [1,11].

Ultrasonography is not routinely used in the evaluation of skewfoot deformity but may help differentiate skewfoot from metatarsus adductus in patients older than three months [9].

Both conservative and surgical treatment options were described. In the absence of symptoms and functional limitations, a watchful waiting approach with regular follow-up could be appropriate. However, the debate remains open as to whether an asymptomatic skewfoot should be treated pre-emptively to prevent future symptoms [16,26]. In symptomatic patients, treatment selection should be based on patient age, symptom severity, clinical presentation, deformity stiffness, and radiographic findings [3,16]. Among these, deformity stiffness is the most critical determinant, as it is strongly influenced by patient age and clinical background. During infancy, the deformity tends to be more flexible, whereas it becomes increasingly rigid once ambulation is established [30]. Furthermore, congenital skewfoot associated with syndromes, as well as iatrogenic skewfoot, is generally more rigid, further complicating treatment decisions [8].

Flexible deformities may be managed with manipulation and serial casting, but there is no consensus on the optimal duration of conservative treatment. Casting duration varies from 3 weeks [30] to 14 weeks [16], and it has been observed that complex skewfoot requires approximately twice the duration (about 26 weeks) of simple skewfoot [16]. Although described by some authors, the use of a Denis Browne bar after serial casting appears to exacerbate hindfoot valgus [16,31,47]; thus, it is not recommended.

Surgery may involve both bone and soft tissue procedures.

Soft tissue procedures, including medial capsulotomy of the navicular–cuneiform and cuneiform–first metatarsal joints, frequently combined with abductor hallucis lengthening [27], improve local balance and enhance the effects of bony realignment, with good results if performed in young children, up to age 6 [44]. Once the compressive forces across the physis are removed, the tarsal bones remodel, grow at a more normal rate, and correct the deformity, in accordance with the Hueter–Volkmann law [48]. Extensive posteromedial release procedures, including talocalcaneal, talonavicular, and navicular-cuneiform capsulotomies, along with Achilles and tibialis posterior lengthening, have also been performed to correct soft tissue imbalance and associated equinus deformity [8].

Bone procedures were frequently employed alongside soft tissue interventions. Medial cuneiform opening wedge osteotomy was the most commonly performed to realign the medial column [22,23,24,25,28]. This procedure targets the apex of deformity, and it is often combined with cuboid closing wedge osteotomy [23,25] or with calcaneal lengthening [28]. The combined approach of osteotomy, peroneus tendon transfer, plantar fascia release, and tibialis posterior advancement [22] or tibialis anterior retention was also described [24]. In cases where initial surgical management failed and for patients with low functional demands due to neurological disease [8], or in cases of subtalar osteoarthritis [30], subtalar or triple fusion may be considered [29,30].

## 5. Conclusions

This scoping review highlights the limited evidence in the current literature about skewfoot. The incidence of skewfoot is likely underestimated due to diagnostic challenges. Skewfoot is often associated with systemic and neurological disorders and, when isolated, may be idiopathic or iatrogenic. Clinical presentation includes the association of forefoot adduction and hindfoot valgus, which can be assessed through conventional weight-bearing radiographs.

Currently, no standardized treatment algorithm exists. Conservative approaches may be applied for flexible feet. Various surgical techniques were reported. Out of these, medial cuneiform opening wedge osteotomy is commonly performed, associated with soft tissue rebalancing procedures. Future studies should aim to accurately standardize diagnostic parameters and treatments in order to establish evidence-based guidelines.

## Figures and Tables

**Table 1:** Main findings from the included studies.

Authors-Years	Study Design and LOE	Number of Feet (n. Patients)	Age at Treatment (Y)	Gender F:M	Etiology	Clinical Presentation	Imaging Evaluation	Finding on Imaging	Intervention
Behan M. et al.-2022 [13]	Case report; LOE V	1 (1)	19	0:1	Iatrogenic following surgical treatment for clubfoot (Achilles tendon release, posteromedial release, and triple fusion)	Hindfoot valgus, midfoot supination, forefoot adduction, medially skewed toes with fixed flexion remnants of clubfoot	X-ray	DP: hindfoot valgus; midfoot supination; forefoot adduction. LL: longitudinal arch flattening.	Surgical: calcaneal dome opening wedge osteotomy with lateral bone block; dorsiflexing osteotomy of the first metatarsal; corrective first metatarsophalangeal joint fusion; and flexor tenotomies of lesser toes and trans fixation with K-wires.
Akimau P. et al.-2014 [22]	Retrospective case series; LOE V	1 (1)	-	-	-	-	X-ray	-	Surgical: lateral column lengthening procedure with ‘a la carte’ bony and soft tissue procedures (calcaneal osteotomy, medial cuneiform osteotomy with iliac crest tricortical bone grafting, peroneus transfer, plantar fascia release, and tibialis posterior advancement)
Kaissi A.A. et al.-2011 [15]	Case report; LOE V	2 (1)	3	0:1	Congenital associated with Freeman–Sheldon Syndrome	Severe forefoot adduction, lateral subluxation of talonavicular joint, hindfoot valgus	X-ray, CT scan	LL: increased talocalcaneal angle; dorsiflexion talonavicular joint and plantar flexion of tarsometatarsal joints. CT: line drawn to the head of the talus and through the body of the talus, making a Z shape.	Conservative: stretching and serial casting.
Hirose C.B. et al.-2004 [23]	Retrospective case series; LOE V	1 (1)	15	0:1	-	-	X-ray	-	Surgical: closing wedge cuboid osteotomy and medial cuneiform opening wedge osteotomy.
Napiontek M. et al.-2003 [24]	Retrospective case series; LOE V	1 (1)	3.8	0:1	-		X-ray	-	Surgical: opening wedge osteotomy of the medial cuneiform and retension of tibialis anterior.
Gordon 2003 [25]	Prospective case series LOE IV;	4 (-)	5.5	-	-	-	X-ray	-	Surgical: closing wedge cuboid osteotomy and medial cuneiform opening wedge osteotomy.
Mirzayan, R et al.-2000 [26]	Case series LOE IV	3 (2)	1.75 (1.5–2)	1:1	Congenital associated with Type I A osteogenesis imperfecta	-	X-ray	DP: metatarsal adductus; lateral subluxation of the navicular on the talus; increase talocalcaneal angle LL: dorsolateral subluxation of the navicular; increased talocalcaneal angle.	No treatment.
Asirvatham R. et al.-1997 [27]	Retrospective case series; LOE IV	17 (-)	3.6 (0.75–5.5)	-	-	-	X-ray	DP: talocalcaneal angle 38° (range, 21°–80°); talo-first metatarsal angle 20° (4°–36°); medial angulation of the base of the middle three metatarsals. LL: talocalcaneal angle 54° (28°–63°).	Surgical: medial capsulotomy of navicular cuneiform and cuneiform first metatarsal and abductor hallucis lengthening.
Mosca V.S. et al.-1995 [28]	Retrospective case series; LOE IV	2 (1)	13.3	-	Congenital idiopathic	Hindfoot valgus, forefoot adduction, retraction of Achilles tendon	X-ray	LL: talo-first metatarsal angle 24° (23°–25°); talo-horizontal angle 40.5° (43°–38°); calcaneal pitch 10° (9°–11°).	Surgical: Opening Wedge Osteotomy of the medial cuneiform, Calcaneal lengthening; Achilles tendon lengthening
		4 (2)	5.75	-	Congenital associated with myelomeningocele	Hindfoot valgus, forefoot adduction, retraction of Achilles tendon	X-ray	LL: talo-first metatarsal angle 28° (3°–52°); talo-horizontal angle 45.5° (25°–65°); calcaneal pitch 4.75° (−7°–14°).	Surgical: opening wedge osteotomy of the medial cuneiform, calcaneal lengthening, Achilles tendon lengthening, talonavicular plication, and tibialis posterior advance.
Scully S.P. et al.-1993 [29]	Case report; LOE V	2 (1)	5	1:0	Congenital associated with Angelman’s syndrome	Severe forefoot adduction, fixed midfoot supination, inflexible hindfoot valgus	X-ray	DP: hindfoot valgus with Kite angle 42°; lateral navicular subluxation; forefoot adduction.	Conservative: serial casting for 3 month and straight shoes. Surgical: plantar fascia release, calcaneal cuboid closing wedge osteotomy and fusion, and metatarsal osteotomies.
		1 (1)	9	1:0	Congenital associated with Angelman’s syndrome	Stiff Forefoot adduction, stiff midfoot supination, stiff hindfoot valgus	X-ray	DP: hindfoot valgus with Kite angle 56°; lateral navicular subluxation; marked forefoot adduction.	Conservative: straight shoes. Surgical at skeletal maturity: triple fusion.
Berg E.E. et al.-1986 [16]	Prospective case series; LOE IV	4 (-)	0.65	-	-	Forefoot adduction, hindfoot valgus	X-ray	DP: talo-first metatarsal angle 16.1°.	No treatment.
		12 (-)	0.49			Forefoot adduction, hindfoot valgus	X-ray	DP: talo-first metatarsal angle 18.5°.	Conservative: serial cast and Denis Brown splints for 7 weeks (1–14).
		4 (-)	0.79			Forefoot adduction, Midfoot lateral translation, hindfoot valgus	X-ray	DP: talo-first metatarsal angle 19.0°.	No treatment.
		11 (-)	0.44	-	-	Forefoot adduction, Midfoot lateral translation, hindfoot valgus	X-ray	DP: talo-first metatarsal angle 13.9°.	Conservative: serial casting and Denis Brown splints for 7.3 weeks (3–12).
Peterson H.A. et al.-1986 [30]	Retrospective case series; LOE IV	2 (1)	0.5	0:1	-	Forefoot adduction, hindfoot valgus	X-ray	DP: increased Kite’s angle; lateral subluxation of navicular bones; metatarsal adduction. LL: increased Kite’s angle.	Conservative: corrective serial cast for 4 week and open-toe outflare shoes for 4 months.
		2 (1)	1.83	1:0	-	Forefoot adduction, hindfoot valgus	X-ray	DP: increased Kite’s angle; lateral subluxation of navicular bones; metatarsal adduction. LL: increased Kite’s angle.	Conservative: corrective serial cast for 3 weeks and open-toe outflare shoes. Surgical: Extensive posteromedial release (talocalcaneal, talonavicular and navicular cuneiform capsulotomy, Achilles and tibialis posterior lengthening), Grice
		4 (2)	2.79 (2.58–3)	1:1	-	Forefoot adduction, hindfoot valgus	X-ray	DP: increased Kite’s angle; lateral subluxation of navicular bones; metatarsal adduction. LL: increased Kite’s angle.	Conservative: corrective open-toe outflare shoes and Denis Browne splints. Surgical: Extensive posteromedial release (talocalcaneal, talonavicular, and navicular cuneiform capsulotomy and Achilles and tibialis posterior lengthening); Grice.
Mean			3.01						
Total		78		5:7					

Abbreviations: LOE—Level of Evidence; Y—years; F—female; M—male; DP—dorsoplantar view in radiograph; LL—lateral view in radiograph; CT—computed tomography.

**Table 2:** Etiology of skewfoot.

Etiology	Subcategory	Details	References
	Associated with systemic disorders	3 cases of osteogenesis imperfecta	Mirzayan, R et al.-2000 [26]
Congenital		3 cases of Angelman’s syndrome	Scully S.P. et al.-1993 [29]
		2 cases of Freeman–Sheldon syndrome	Kaissi A.A. et al.-2011 [15]
	Associated with neurogenic conditions	4 cases with myelomeningocele	Mosca V.S. et al.-1995 [28]
Idiopathic		2 cases	Mosca V.S. et al.-1995 [28]
Iatrogenic		1 case following surgical treatment for clubfoot (Achilles tendon release, posteromedial release, and triple fusion)	Behan M. et al.-2022 [13]
Not reported		55 cases	Akimau P. et al.-2014 [22] Hirose C.B. et al.-2004 [23] Napiontek M. et al.-2003 [24] Gordon 2003 [25] Asirvatham R. et al.-1997 [27] Berg E.E. et al.-1986 [16] Peterson H.A. et al.-1986 [30]

**Table 3:** Clinical and imaging presentation of skewfoot.

Authors-Years	Clinical Presentation	Finding on Imaging
Behan M. et al.-2022 [13]	Hindfoot valgus; midfoot supination; forefoot adduction; medially skewed toes with fixed flexion remnants of clubfoot.	DP: hindfoot valgus; midfoot supination; forefoot adduction. LL: longitudinal arch flattening.
Akimau P. et al.-2014 [22]	-	-
Kaissi A.A. et al.-2011 [15]	Severe forefoot adduction; lateral subluxation of talonavicular joint; hindfoot valgus.	LL: increased talocalcaneal angle; dorsiflexion talonavicular joint and plantar flexion of tarsometatarsal joints. CT: a line drawn to the head of the talus and through the body of the talus forming a Z shape.
Hirose C.B. et al.-2004 [23]	-	-
Napiontek M. et al.-2003 [24]		-
Gordon-2003 [25]	-	-
Mirzayan, R et al.-2000 [26]	-	DP: metatarsal adductus; lateral subluxation of the navicular on the talus; increase talocalcaneal angle. LL: dorsolateral subluxation of the navicular; increased talocalcaneal angle.
Asirvatham R. et al.-1997 [27]	-	DP: talocalcaneal angle 38° (range; 21°–80°); talo-first metatarsal angle 20° (4°–36°); medial angulation of the base of the middle three metatarsals. LL: talocalcaneal angle 54° (28°–63°)
Mosca V.S. et al.-1995 [28]	Hindfoot valgus; forefoot adduction; retraction of Achilles tendon.	LL: talo-first metatarsal angle 24° (23°–25°); talo-horizontal angle 40.5° (43°–38°); calcaneal pitch 10° (9°–11°).
		LL: talo-first metatarsal angle 28° (3°–52°); talo-horizontal angle 45.5° (25°–65°); calcaneal pitch 4.75° (7°–14°).
Scully S.P. et al.-1993 [29]	Severe forefoot adduction; fixed midfoot supination; inflexible hindfoot valgus.	DP: hindfoot valgus with Kite angle 42°; lateral navicular subluxation; forefoot adduction.
	Stiff Forefoot adduction; stiff midfoot supination; stiff hindfoot valgus.	DP: hindfoot valgus with Kite angle 56°; lateral navicular subluxation; marked forefoot adduction.
Berg E.E. et al.-1986 [16]	Forefoot adduction; hindfoot valgus.	DP: talo first metatarsal angle 16.1°.
	Forefoot adduction; hindfoot valgus.	DP: talo first metatarsal angle 18.5°.
	Forefoot adduction; Midfoot lateral translation; hindfoot valgus.	DP: talo first metatarsal angle 19.0°.
	Forefoot adduction; Midfoot lateral translation; hindfoot valgus.	DP: talo first metatarsal angle 13.9°.
Peterson H.A. et al.-1986 [30]	Forefoot adduction; hindfoot valgus.	DP: increased Kite’s angle; lateral subluxation of navicular bones; metatarsal adduction. LL: increased Kite’s angle.

**Table 4:** Treatment options.

Treatment	Procedure	Cases (%)	Mean Age (Years)	References
Conservative	• Gentle manipulations, stretching, and serial casting in corrective positions	33 (42%)	2.5	Kaissi A.A. et al.-2011 [15]
			(0.5–2.75)	Berg E.E. et al.-1986 [16]
				Scully S.P. et al.-1993 [29], Peterson H.A. et al.-1986 [30]
	• Denis Browne splints	27 (35%)	—	Berg E.E. et al.-1986 [16], Peterson H.A. et al.-1986 [30]
	• Outflare shoes	8 (10%)	—	Peterson H.A. et al.-1986 [30]
	• Straight shoes	3 (4%)	—	Scully S.P. et al.-1993 [29]
Surgical-Soft Tissue	• Medial capsulotomy of the navicular–cuneiform and cuneiform–first metatarsal joints + abductor hallucis lengthening	17 (22%)	3.6	Asirvatham R. et al.-1997 [27]
			(0.75–5.5)	
	• Extensive posteromedial release + Achilles and tibialis posterior tendon lengthening	6 (8%)	—	Peterson H.A. et al.-1986 [30]
Surgical-Bone	• Medial cuneiform opening wedge osteotomy	13 (17%)	7.5 (3.8–15)	Hirose C.B. et al.-2004 [23], Akimau P. et al.-2014 [22], Gordon 2003 [25]
				Mosca V.S. et al.-1995 [28]
				Napiontek M. et al.-2003 [24]
	• + Cuboid closing wedge osteotomy	5 (6%)	7.4 (5.5–15)	Hirose C.B. et al.-2004 [23], Gordon 2003 [25]
	• + Calcaneal lengthening and Achilles tendon lengthening	6 (8%)	8.3 (5.75–13)	Mosca V.S. et al.-1995 [28]
	• + Talonavicular plication and tibialis posterior advancement	4 (5%)	—	Mosca V.S. et al.-1995 [28]
	• + Calcaneal osteotomy, peroneus transfer, plantar fascia release, and tibialis posterior advancement	1 (1%)	—	Akimau P. et al.-2014 [22]
	• Tibialis anterior retensioning	1 (1%)	3.8	Napiontek M. et al.-2003 [24]
	• Calcaneal cuboid closing wedge fusion + multiple metatarsal osteotomies + plantar fascia release	2 (3%)	5	Scully S.P. et al.-1993 [29]
	• Calcaneal dome lateral opening wedge osteotomy + dorsiflexing 1st metatarsal osteotomy + MTP fusion + K-wire fixation	1 (1%)	—	Behan M. et al.-2022 [13]

## Data Availability

No new data was created, or data is unavailable due to privacy or ethical restrictions.
